# Putting Beta-Diversity on the Map: Broad-Scale Congruence and Coincidence in the Extremes

**DOI:** 10.1371/journal.pbio.0050272

**Published:** 2007-10-09

**Authors:** Meghan W McKnight, Peter S White, Robert I McDonald, John F Lamoreux, Wes Sechrest, Robert S Ridgely, Simon N Stuart

**Affiliations:** 1 Curriculum in Ecology, University of North Carolina at Chapel Hill, Chapel Hill, North Carolina, United States of America; 2 Department of Biology, University of North Carolina at Chapel Hill, Chapel Hill, North Carolina, United States of America; 3 Graduate School of Design, Harvard University, Cambridge, Massachusetts, United States of America; 4 IUCN/SSC–CI/CABS Biodiversity Assessment Unit, Conservation International, Arlington, Virginia, United States of America; 5 Department of Wildlife and Fisheries Sciences, Texas A&M University, College Station, Texas, United States of America; 6 Department of Environmental Sciences, University of Virginia, Charlottesville, Virginia, United States of America; 7 World Land Trust–US, Deerfield, New Hampshire, United States of America; Imperial College London, United Kingdom

## Abstract

Beta-diversity, the change in species composition between places, is a critical but poorly understood component of biological diversity. Patterns of beta-diversity provide information central to many ecological and evolutionary questions, as well as to conservation planning. Yet beta-diversity is rarely studied across large extents, and the degree of similarity of patterns among taxa at such scales remains untested. To our knowledge, this is the first broad-scale analysis of cross-taxon congruence in beta-diversity, and introduces a new method to map beta-diversity continuously across regions. Congruence between amphibian, bird, and mammal beta-diversity in the Western Hemisphere varies with both geographic location and spatial extent. We demonstrate that areas of high beta-diversity for the three taxa largely coincide, but areas of low beta-diversity exhibit little overlap. These findings suggest that similar processes lead to high levels of differentiation in amphibian, bird, and mammal assemblages, while the ecological and biogeographic factors influencing homogeneity in vertebrate assemblages vary. Knowledge of beta-diversity congruence can help formulate hypotheses about the mechanisms governing regional diversity patterns and should inform conservation, especially as threat from global climate change increases.

## Author Summary

Beta-diversity—how species composition varies from place to place—is a fundamental attribute of biodiversity. However, despite its recognized importance, beta-diversity is rarely studied across large spatial scales. Here we use a new method to compare amphibian, bird, and mammal beta-diversity across large regions within the Western Hemisphere. We show that although the areas of low beta-diversity are different for the three groups, areas of high beta-diversity largely coincide. Moreover, we find that the degree to which the groups exhibit similar patterns of beta-diversity depends on the geographic location and extent at which it is measured. Beta-diversity is high where species are most susceptible to climate change, such as in areas with complex topography or high environmental variation. Identifying where areas of high beta-diversity coincide for different species groups is essential to the design of effective protected area networks.

## Abstract 3

A new method to compare amphibian, bird, and mammal beta-diversity within the Western Hemisphere reveals that areas of high beta-diversity coincide, with implications for understanding regional diversity patterns and for conservation.

## Introduction

Beta-diversity, the change in species composition between places, represents the differentiation component of diversity, as opposed to the inventory component, which describes the species composition of a single place [1–3]. Although beta-diversity was originally defined as the differentiation of communities along environmental gradients [1], the concept applies more widely to the phenomenon of species compositional change at any scale, regardless of mechanism [2–7]. Beta-diversity *sensu lato* is determined through a complex array of processes relating to the interaction of species traits (e.g., vagility and niche width) and characteristics of the physical landscape (e.g., environmental dissimilarity, topographic complexity, and isolation) over time [3,8–11]. Geographic variation in beta-diversity, from gradual changes to abrupt transitions, reflects past and present differences in environment, ecological interactions, and biogeographic history, including barriers to dispersal [4,7,9–15].

As beta-diversity quantifies the change, or turnover, in species across space, it is central to a wide array of ecological and evolutionary topics, such as the scaling of diversity [16–19], the delineation of biotic regions or biotic transitions [20,21], and the mechanisms through which regional biotas are formed [15,20–22]. Beta-diversity also provides information critical to conservation planning, which strives to represent all biodiversity within practical constraints such as area and cost [11,14,16,23,24]. While the total number of species, endemic species, or threatened species often contributes to the relative importance of an area [25–29], it is the rate of species turnover between sites that dictates the optimal spatial arrangement of conservation areas [10,11,16]. Although the principles behind most approaches to systematic planning, such as complementarity, are driven by patterns of beta-diversity [23,30], few methods make explicit use of turnover measures [6,31]. Directly incorporating beta-diversity patterns into priority setting, however, benefits conservation efforts. For example, modeling compositional dissimilarity to develop surrogates for data-poor regions can improve biodiversity representation [6,30,32]. Moreover, including turnover estimates in area selection algorithms captures variation in species assemblages, which helps to preserve ecological and evolutionary processes as well as underlying environmental heterogeneity necessary for long-term persistence [28,31].

Despite the importance of beta-diversity, relatively little is known about diversity's “other component,” particularly at broad scales. This is largely because measures of beta-diversity require knowledge of species identities rather than just species counts. Recent advances in species distributional data have made beta-diversity analyses possible at large extents [17,20,21], but these studies have been limited to single taxa. Cross-taxon congruence in beta-diversity has been tested only at small scales, with varying methods and results [14,22,32,33], in contrast to the wide range of scales at which concordance in both species richness and endemism has been studied [34–38]. Here, we present what is to our knowledge the first analysis of beta-diversity congruence across large spatial scales, based on distributional data for three groups of terrestrial vertebrates in the continental Western Hemisphere.

Beta-diversity of amphibians (*n* = 2,174) [39], breeding birds (*n* = 3,882) [40], and mammals (*n* = 1,611) [41] was estimated as a function of the distance decay of similarity—the decrease in compositional similarity with increasing geographic distance between sites [4,7,10,14]. We modeled distance decay from each 100 km × 100 km grid cell, and used these models to calculate our measure of beta-diversity, β_sim-d_: the estimated proportional turnover in species composition at a distance of 100 km (see Materials and Methods). This individual-cell-based technique accounts for the considerable geographic variation in the rate at which similarity decays, and can be used to produce a continuous layer of compositional change similar to past grid-based neighborhood analyses of broad-scale beta-diversity (e.g., [18,20,21]). Considering comparisons over a range of distances reduces possible bias in similarity levels that could arise from the differences in centroid to centroid distance and in shared perimeter length that occur between orthogonal and diagonal neighbors of a rectangular grid. The smoothing that results from the distance decay regressions also limits the influence of artifacts due to small-scale errors in range map boundary placement.

Our approach to quantifying the distance decay relationship makes several improvements to methods used in previous studies [4,7,10,14]. For instance, we modeled distance decay using logistic regression, which has advantages over linear or log-linear ordinary least-square regressions [4,7,14], particularly for proportional data [42]. Furthermore, following Lennon et al. [18], we measured similarity with a metric shown to be independent of differences in species richness between grid cells in order to isolate change due to species replacement [43] (see Materials and Methods).

We tested congruence in β_sim-d_ for the three taxa using two different approaches. With the first, we measured congruence in overall β_sim-d_ patterns and examined whether congruence levels were consistent across multiple spatial extents and among different geographic locations. In the second approach, we quantified spatial overlap in the extremes of β_sim-d_. We report that the strength of congruence depends on the location and extent at which it is measured, and that overlap in high β_sim-d_ is much greater than in low β_sim-d_. Furthermore, the pairs of taxa varied substantially in level of congruence and degree of overlap.

## Results/Discussion

### β_sim-d_ Patterns

**Figure 1:**
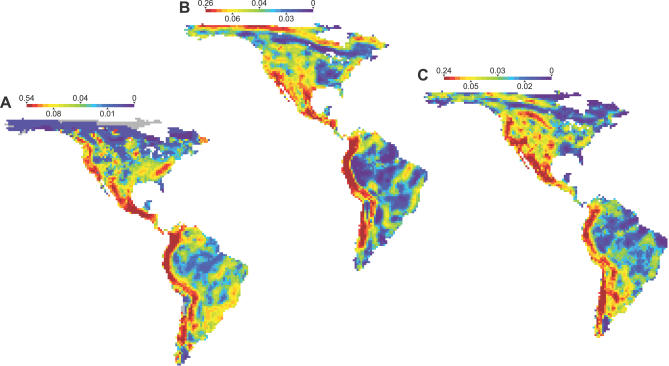
Beta-Diversity of Amphibians, Birds, and Mammals Mapped Continuously across the Continental Western Hemisphere Beta-diversity (β_sim-d_) values for each taxon are divided into 20 quantiles, represented by warm (higher β_sim-d_) to cool (lower β_sim-d_) colors. The scale accompanying the color ramp for each taxon shows minimum, first quartile, median, third quartile, and maximum values of β_sim-d_. Gray grid cells do not contain amphibian species. (A) Amphibians. (B) Birds. (C) Mammals.

Amphibian, bird, and mammal β_sim-d_ mapped at this scale (Figure 1) provide a striking contrast to well-known patterns of broad-scale species richness for these vertebrate groups. Whereas high richness is generally concentrated in the tropics and decreases towards both poles [44], β_sim-d_ of all levels is found across a wide range of latitudes. High β_sim-d_ stretches along the mountainous Pacific edge of the continents, while low β_sim-d_ is found within more environmentally uniform portions of northern South America and boreal North America. Accordingly, β_sim-d_ has a positive relationship with both elevation and number of biome boundaries (β_sim-d_ and elevation: Spearman rank ρ = 0.219–0.427, *p <* 0.05 for amphibian β_sim-d_, *p <* 0.001 for other taxa; β_sim-d_ and biome edge: ρ = 0.295–0.320, *p <* 0.001 for all; Table S1; see Materials and Methods). Although the variables show considerable spread (Figure S1), high β_sim-d_ grid cells of all three groups occur at significantly higher elevations and on a greater number of biome edges than expected by chance alone, while low β_sim-d_ grid cells have significantly lower elevations and fewer biome edges than expected by chance (Table S2; 10,000 random sets, *p <* 0.05 for elevation in amphibian low β_sim-d_ grid cells, *p <* 0.001 for all others; see Materials and Methods). The weaker significance for elevation in amphibian low β_sim-d_ grid cells is likely due to the wood frog (Rana sylvatica) being the only amphibian species to occur throughout much of the boreal region, including high-altitude areas such as the Alaska panhandle [45]. This amphibian homogeneity differs greatly from the high β_sim-d_ of birds at northern latitudes, which captures the presence of a strong Holarctic element in the avifauna along the arctic coast [46]. Such differences in β_sim-d_ reveal the individual biogeographic histories of the taxa and may arise from variation in dispersal ability, particularly in relation to historical factors such as glaciation and faunal interchange [45,47]. For instance, the elevated mammal β_sim-d_ in South America's southern cone reflects a transition in the region's diverse mammal lineages, notably the radiation of narrowly ranging hystricognath rodents [48], while the high amphibian β_sim-d_ of the southern Appalachian Mountains results from the diversification of salamanders within this area's stable, moist environments [45].

### Congruence in Overall β_sim-d_ Patterns

**Figure 2:**
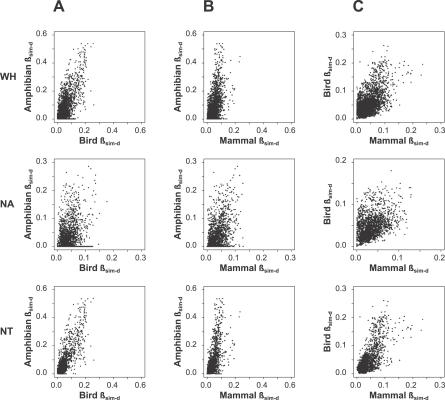
Cross-Taxon Relationships in Beta-Diversity of Amphibians, Birds, and Mammals Scatter plots show the relationships between β_sim-d_ for each pair of taxa within the Western Hemisphere (WH, top row), the Nearctic realm (NA, middle row), and the Neotropical realm (NT, bottom row). The axes for each plot are scaled according to the maximum β_sim-d_ value of the two taxa within the extent specified. Note that maximum values are much greater for amphibians than for either birds or mammals and that all three taxa reach higher rates of assemblage change in the Neotropics than in the Nearctic. (A) Amphibians and birds. (B) Amphibians and mammals. (C) Birds and mammals.

Pair-wise correlations of amphibian, bird, and mammal β_sim-d_ across the Western Hemisphere were positive and significant (ρ = 0.340–0.553, *p <* 0.001 for all; see Materials and Methods) (Table 1; Figure 2). When measured at the extent of a single biogeographic realm, however, we found that pair-wise congruence was greater within the Neotropics (ρ = 0.636–0.695, *p <* 0.001 for all) than at the hemisphere extent, but was comparatively weak within the Nearctic (amphibians and mammals: ρ = 0.390, *p <* 0.05; birds and mammals: ρ = 0.405, *p <* 0.001) or even lacking (amphibians and birds: ρ = 0.032, not significant) (Table 1; Figure 2). The disparity in congruence strength between the realms indicates that congruence measured across large regions can hide incongruities that manifest at reduced spatial extents [35,36].

**Table 1:**
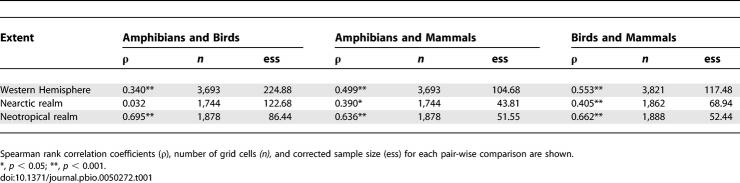
Correlations in Beta-Diversity (β_sim-d_) within the Western Hemisphere, Nearctic Realm, and Neotropical Realm

**Figure 3:**
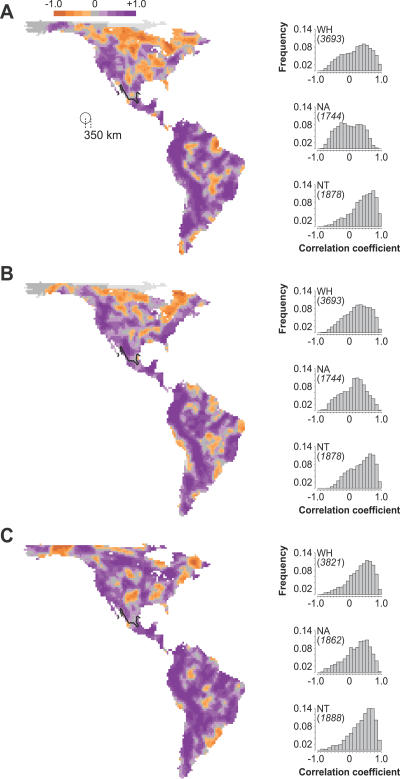
Geographic Variation in Beta-Diversity Congruence of Amphibians, Birds, and Mammals at Small Spatial Extents The color of each grid cell indicates the strength of beta-diversity (β_sim-d_) congruence calculated within a 350-km-radius window around that grid cell. Orange shades represent strong (darkest) to weak (lightest) negative correlations. Purple shades show strong (darkest) to weak (lightest) positive correlations. Dark gray indicates very weak correlations of either sign, or no correlation. Light gray grid cells do not contain amphibian species. Shown to the right of each map are frequency distributions of correlation coefficients for windows located within the entire Western Hemisphere (WH), the Nearctic realm (NA), and the Neotropical realm (NT), which are consistent with the overall level of congruence measured at these extents. The black line marks the boundary between the two realms. (A) Amphibians and birds. (B) Amphibians and mammals. (C) Birds and mammals.

To examine congruence at even smaller extents, we used a moving-window algorithm that calculated the correlation in β_sim-d_ between each pair of taxa within a 350-km radius of each grid cell (see Materials and Methods). Composite maps of the resulting correlation coefficients for the pairs revealed considerable geographic variation in congruence (Figure 3). Although the majority of correlations were strongly positive, others were weak or strongly negative. The latter were most apparent in the Nearctic realm for correlations with amphibians. Understanding the dependence of diversity relationships on observational scale is of pressing concern for ecology, biogeography, and conservation planning [10,18,23,36]. Our analyses demonstrate that both the geographic location and the spatial extent of analysis affect the level of congruence observed in β_sim-d_, and emphasize the need for tests across multiple scales and regions in order to make objective comparisons among ecological studies.

### Spatial Overlap in High and Low β_sim-d_

Correlations across all grid cells do not necessarily indicate the level of cross-taxon spatial coincidence in areas of highest or lowest β_sim-d_—a more useful measure for conservation planning and biogeographic delineation [34,35,49]. Congruence in the extremes of diversity is frequently measured as the degree of overlap in matching percentage sets of two groups [34,50]. We evaluated high and low β_sim-d_ congruence for the pairs of taxa and between all three groups as the proportion of maximum possible overlap [34] in matching percentage sets of the highest 2.5% and lowest 2.5% of each taxon's β_sim-d_ grid cells (see Materials and Methods).

**Figure 4:**
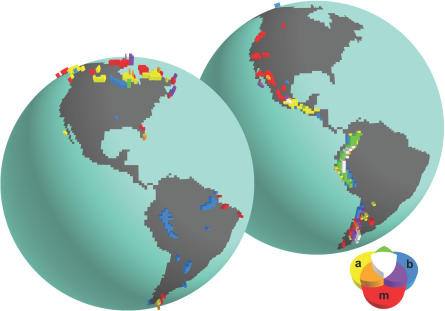
Geographic Distribution of Amphibian, Bird, and Mammal High and Low Beta-Diversity Overlap Spatial overlap in beta-diversity (β_sim-d_) for percentage sets of each taxon's lowest (left) and highest (right) 2.5% of β_sim-d_ grid cells is shown. Primary colors indicate grid cells unique to one taxon (yellow, amphibians; blue, birds; red, mammals), secondary colors indicate overlap between two groups, and white indicates overlap of all three groups. The height of the grid cells reflects the number of overlapping groups. Note the greater degree of spatial coincidence in high β_sim-d_ than in low β_sim-d_.

Spatial coincidence in high β_sim-d_ was greatest between amphibians and birds (51.6%). These taxa showed lower, but similar levels of overlap in high β_sim-d_ with mammals (21.5% and 29.2%, respectively), and coincidence between all three groups was minimal (15.1%). Grid cells with overlapping high β_sim-d_ primarily occurred in the northern and southern Andes (Figure 4), consistent with the former as a center of endemism for all three taxa and with the extreme climatic gradient within the latter [44,45]. A substantial proportion of grid cells were found only in the high β_sim-d_ percentage sets of one taxon. For example, 41.9% of amphibian high β_sim-d_ grid cells were unique, as were 35.4% of bird high β_sim-d_ grid cells and 64.6% of mammal high β_sim-d_ grid cells. The distribution of these grid cells reflects the specific biogeographies of each taxon. Whereas unique grid cells were predominantly located in the northern Andes for birds and in the Central American highlands for amphibians, unique mammal grid cells were largely outside the tropics (Figure 4).

There was comparatively little spatial coincidence in the lowest 2.5% of β_sim-d_. Low β_sim-d_ of birds and mammals showed the most overlap, at only 11.5%. Coincidence was negligible for the other two pairs of taxa (amphibians and mammals, 5.4%; amphibians and birds, 2.2%), and there was no overlap among all three groups. Accordingly, the majority of grid cells in the low β_sim-d_ percentage sets were restricted to one taxon (83.3%–92.5%). These grid cells were located mainly in the boreal and arctic regions of the Nearctic realm for amphibians and mammals, respectively (Figure 4). Conversely, most unique bird grid cells occurred in the Neotropics within several biomes, including a substantial number in the Amazon Basin (Figure 4).

**Figure 5:**
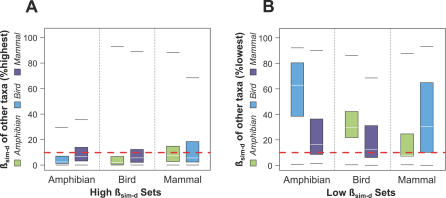
Levels of Beta-Diversity for Vertebrate Taxa within Areas of High and Low Beta-Diversity of Amphibians, Birds, and Mammals Percentage sets of the highest (A) and lowest (B) 2.5% of beta-diversity (β_sim-d_) grid cells for one taxon (*x*-axis) contain a range of β_sim-d_ levels for the other taxa (*y*-axis), as shown by the box plots (white lines within boxes indicate the median; top and bottom box edges indicate first and third quartiles; black lines indicate minimum and maximum percentage rank of β_sim-d_). The red dashed line indicates the highest or lowest 10% of β_sim-d_.

The degree of overlap in matching percentage sets, however, does not provide a complete picture of spatial coincidence in the extremes of β_sim-d_. In fact, the majority of highest β_sim-d_ grid cells for all three taxa actually had relatively high levels of β_sim-d_ for the other groups (Figure 5), indicating that areas of high beta-diversity largely coincide. On average, more than two-thirds of grid cells in the highest 2.5% of one taxon's β_sim-d_ grid cells were also in the highest 10% of β_sim-d_ for the other taxa (70.0% ± 8.7%, range = 61.5%–81.7%). This was not true for low β_sim-d_. Low β_sim-d_ grid cell sets exhibited greater variation in β_sim-d_ values for the other taxa than did the high β_sim-d_ sets. Moreover, less than one-quarter of the lowest 2.5% of one taxon's β_sim-d_ grid cells were in the lowest 10% of β_sim-d_ for the other taxa (21.9% ± 14.6%, range = 2.9%–40.6%)—further evidence that areas of low β_sim-d_ are spatially distinct (Figure 5).

### Conclusions

Congruence in beta-diversity of three groups of terrestrial vertebrates is highly dependent on the geographic location and extent of analysis, reflecting taxonomic and regional variation in the influence of large-scale historical processes and environmental factors [4,7,10,14,15]. Our results show that although correlations in amphibian, bird, and mammal β_sim-d_ measured at small extents vary in strength throughout the Western Hemisphere, congruence is generally stronger within the Neotropical realm than within the Nearctic. This difference may be part of a broader asymmetry in biodiversity patterns between the Northern Hemisphere and the Southern Hemisphere [51,52]. The weak pair-wise correlations within the Nearctic realm, as well as the minimal overlap in both high and low β_sim-d_, could result from differing responses of amphibians, birds, and mammals to the realm's climatic and geologic history [45,47]. In contrast, the comparatively strong β_sim-d_ congruence in the Neotropics is indicative of common patterns of speciation and extinction histories. This is particularly apparent within the Neotropical mountains, where the substantial overlap in high β_sim-d_ among the three groups underscores the importance of this region in generating diversity. Variation in β_sim-d_ congruence also has implications for conservation, because the efficacy of conservation surrogates and efforts to model overall biodiversity distribution depend on taxa having concordant patterns of compositional change [30]. Our results largely support these approaches, but it is important to recognize limitations that may arise from differing congruence levels among biogeographic realms.

Regions of rapid species turnover require increased attention to the placement and size of conservation areas in order to protect biodiversity. Spatial coincidence in areas of high β_sim-d_ is therefore encouraging, as successful conservation strategies in these places may be resource intensive. Conservation planning, of course, must occur across hierarchical scales in order to ensure adequate representation [23,28]. Broad-scale analyses of β_sim-d_ highlight regions where protected areas should be closely spaced to effectively conserve biodiversity; however, the optimal configuration for conservation networks will depend on finer-scale beta-diversity patterns [53]. Mapping broad-scale β_sim-d_ can also identify areas where species face increasing threat to persistence. For example, because β_sim-d_ is high where species' ranges are particularly susceptible to climatic variability, such as at steep environmental gradients and centers of endemism [54–56], or at biome transitions where range shifts are most noticeable [54,55], we suggest that areas of high β_sim-d_ are likely to be especially vulnerable to climate change.

The unique biogeography of the Western Hemisphere—the great variation in the effects of Pleistocene glaciation, the complex of mountain chains along much of the western coast, and the relative isolation of the continents—has played a major role in shaping the distribution and evolution of biodiversity. More work is needed to determine if our findings will extend to other parts of the world with different geologic and environmental histories. Furthermore, the relative contribution of historical factors and current ecological interactions in determining beta-diversity patterns and congruence in beta-diversity across taxa is an important area of inquiry.

Our results describe patterns of species turnover at a 100 km × 100 km resolution. As comprehensive finer-resolution data become available, further analyses will confirm whether the levels of beta-diversity and congruence we found are consistent with those measured at smaller grain sizes. Future research is also needed to ascertain the degree to which our results can be generalized to other taxa, especially more distantly related groups or those that show large variation in dispersal ability. For instance, taxa with poor dispersal and low rates of gene flow are apt to exhibit higher beta-diversity than those groups that have high dispersal and high rates of gene flow. However, we believe that some of our findings, such as the strong relationship between topography and beta-diversity congruence, will prove true for most taxa.

## Materials and Methods

###  Data.

Analyses were based on range data for extant species of amphibians (*n* = 2,174), breeding birds (*n* = 3,882), and mammals (*n* = 1,611) in the Western Hemisphere [39–41]. The range maps used for this study were obtained as digital vector files (ArcView format) from the Web sites indicated by [39–41], where one can also find information on updates, detailed descriptions of the production process, and complete lists of sources. Note that these datasets are periodically updated, and the files used for these analyses may differ from the most recent versions available from [39–41]. We confined our analyses to terrestrial breeding birds, and we provide a map of bird β_sim-d_ based on both breeding and non-breeding ranges of all terrestrial birds (*n* = 3,890) for comparison. β_sim-d_ for all birds (Figure S2) was highly correlated with β_sim-d_ for breeding birds (Figure 1) (ρ = 0.954, estimated sample size [ess] = 249.12, *p <* 0.001). The number of species in these vertebrate groups is not static, as new species, especially of amphibians, continue to be discovered [57]. However, the areas from which species are most often described tend to be the same and will likely accentuate the patterns we present [58]. In relation to this point, systematic bias in the data may result from differences in sampling efforts, as the distributions of certain groups (e.g., birds) or geographic areas (e.g., temperate regions) for which sampling efforts have been intense will be more reliable than those that are undersampled (e.g., amphibians or tropical regions). As a precaution against such bias, we excluded from the analyses the 630 amphibian species with an IUCN Red List category of “data deficient” (http://www.redlist.org/) because of the unreliability of their range maps. The exclusion of these species did not substantially affect our results (correlation between amphibian β_sim-d_ using all mapped species and amphibian β_sim-d_ excluding “data deficient” species; ρ = 0.993, ess = 158.6, *p <* 0.001).

We recorded the presence/absence of each species in 100 km × 100 km equal-area grid cells, roughly equivalent to 1° × 1° at the equator (Behrmann projection, WGS84 datum); a species was considered present if any portion of its range (exclusive of polygons coded as introduced, migratory, or vagrant) occurred within the continental land area of the grid cell. However, the range maps used approximate the extent of occurrence of a species, rather than its area of occupancy, and therefore the species may not be found in every grid cell that falls within the mapped range [59,60]. Such biases are inherent in range data compiled across large regions, and remind us that while the patterns they show inform us about species turnover at broad scales, they are not a replacement for finer-scale distributional information.

Grid cells on the perimeter of the continents vary considerably in the amount of land they contain, particularly those along the narrow Isthmus of Panama. To avoid potential effects of species–area relationships or errors from range map boundary placement, only grid cells containing ≥40% of continental land were included in the analyses (grid cells: *n* = 3,693 for amphibians; *n* = 3,821 for birds and mammals). Estimates of β_sim-d_ using this cutoff were not appreciably different from those based on a more conservative cutoff of 75% land area, but allowed for the inclusion of additional species. Grid cells were classified as either Nearctic (*n* = 1,744 for amphibians; *n* = 1,862 for birds and mammals), Neotropical (*n* = 1,878, amphibians; *n* = 1,888, birds and mammals), or transitional between the two biogeographic realms (*n* = 71 for all taxa) [61]. Transitional grid cells were not included in analyses at the realm extent.

### Analyses.

We used a moving-window algorithm to model the distance decay of similarity from each individual grid cell, and used the resulting regression parameters to calculate a value of beta-diversity, β_sim-d_, as the estimated proportional turnover from that grid cell at a distance of 100 km. Considering comparisons between grid cells over a range of distances helps alleviate concerns typical of gridded nearest-neighbor analyses of large-scale species distributions. For example, artifacts may arise from the small-scale errors that can occur in range boundary placement when converting polygon maps into gridded data, as well as from the discrepancy in centroid to centroid distance and shared perimeter length between orthogonal and diagonal neighbors in a rectangular grid.

Similarity (*S*) between two grid cells was calculated as the complement of β_sim_, a dissimilarity metric that isolates change due to species replacement from differences in species richness:

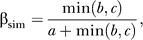
where *a* is the number of species shared, *b* is the number of species found only in the second grid cell, and *c* is the number of species found only in the first grid cell, making min(*b*, *c*) the number of unshared species in the more depauperate grid cell [18,43]. Therefore, as the complement of β_sim_ (i.e., 1 − β_sim_),

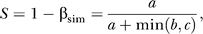
or the proportion of species in the more depauperate grid cell that also occur in the other grid cell.

Note that *S*/1 − *S* is a transformation of the ratio of shared species to unshared species in the more depauperate grid cell, or *a*/min(*b*, *c*). This enables us to model distance decay using a logistic regression functional form defined such that

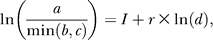
where *d* is the centroid to centroid distance, and *I* and *r* are fitted intercept and slope coefficients. This functional form has an advantage over linear and log-linear forms, in that 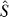 is bound between zero and one, as is appropriate for a similarity index. The observed data are counts, and we use a binomial error distribution for our regression. This has several advantages over linear and log-linear regressions with a normal error distribution, resulting in a better empirical fit than other techniques [42]. First, the special cases where *S* = 0 (i.e., *a* = 0) and *S* = 1 (i.e., min(*b*, *c*) = 0) do not cause problems in the estimation process, as these are valid possibilities under the binomial distribution. Second, the binomial error distribution accounts for the greater variance in *a*/min(*b*, *c*) (and hence *S*) at low species numbers.

The distance decay regression at each window was built using between-grid-cell comparisons of the focal grid cell and all grid cells within a ≤500-km centroid to centroid radius. Thus, unlike most published distance decay regressions, which compute a rate of change based on comparisons between all samples within a region, our regressions are based on comparisons only with the focal grid cell and therefore reflect the change from a particular point (i.e., grid cell). The arbitrary distance of 500 km was chosen after experimenting with several other maximum distances (350, 1,000, 1,500, 2,000, and 3,000 km) because it provided a sufficient total number of between-grid comparisons (i.e., sample size), spread over a range of distances, to ensure a robust distance decay relationship, but did not result in an over-smoothed beta-diversity surface, as occurred with greater maximum distances (as judged by visual comparisons of the maps). By transforming the above defined regression equation as

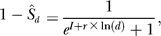
it is possible to use any set of distance decay regression coefficients (*I* and *r*) to predict the proportional dissimilarity at any distance (*d*). Thus, we used the coefficients from the distance decay regression for each grid cell to estimate our measure of beta-diversity, β_sim-d_, as (1 − 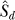 ) for *d* = 100 km, or the estimated proportional turnover in composition from that grid cell at a distance of 100 km.

The predicted degree of dissimilarity at a given distance, β_sim-d_, differs from the average observed dissimilarity (β_sim_) at the same distance because it accounts for the rate at which dissimilarity changes with increasing distance (i.e., the effect of extent). At the same time, β_sim-d_ differs from the rate of distance decay in the following ways. (i) Estimates of β_sim-d_ depend on both the intercept and slope parameters of the distance decay relationship. The former, as initial similarity level, reflects dissimilarity at near distances and the latter, as the rate of distance decay, captures dependency of dissimilarity on extent [62,63]. (ii) β_sim-d_ is the estimated dissimilarity at a specified distance (i.e., 100 km)—predictions at other distances (e.g., 50 or 300 km) would result in different values, reflecting the effect of spatial extent on compositional change. Turnover at this distance, which is the minimum distance between adjacent grid cells, is more intuitive than that between distant grid cells for discussion and graphical representation of beta-diversity as a continuous surface, and makes it easier to compare our results to other broad-scale diversity analyses.

Although the number of grid cells included in a regression model decreased with increased proximity to the coast (including major interior water bodies), graphical examination of scatter plots and the resulting maps showed that coastal effects were negligible for amphibians and mammals and varied geographically for birds. The elevated bird β_sim-d_ on some coastal sections likely has a biological rather than methodological basis [18]. It is important to remember that β_sim-d_ quantifies change in species composition between 100 km × 100 km grid cells, and therefore does not reflect the level of heterogeneity within a grid cell. Furthermore, β_sim-d_ is a measure of proportional species turnover and does not represent the absolute number of species gained or lost between grid cells. Lastly, while the smooth surface that results from modeling the effect of distance on similarity reduces the effect of potential errors in gridded large-scale range data, extremely abrupt transitions may be attenuated. However, the major patterns found for β_sim-d_ were also apparent in maps of average nearest-neighbor beta-diversity (the average dissimilarity [β_sim_] of a focal grid cell and its orthogonal and diagonal neighbors) (Figure S3). Further, a comparison of Table 1 with pair-wise correlations of average β_sim_ (Table S3) shows that the congruence levels we report are not artifacts of the smoothing process.

We tested whether grid cells containing high β_sim-d_ or those with low β_sim-d_ differed significantly in elevation or were found on a greater number of biome edges than could be expected by chance [64]. To do this, we selected sets of grid cells containing the highest 2.5% and the lowest 2.5% of β_sim-d_ values for each taxon (2.5% = 93 grid cells for amphibians, 96 grid cells for birds and mammals), and calculated the mean elevation and mean number of biome edges for each set. We then compared these values to distributions of values for the mean elevation and mean number of biome edges, respectively, calculated for 10,000 sets of randomly selected grid cells (grid cells per random set: *n* = 93 for amphibians; *n* = 96 for birds and mammals). For each comparison, we computed a one-tailed *p*-value by counting the number of values in the random distribution greater than or equal to the value of a high β_sim-d_ set—or less than or equal to the value of a low β_sim-d_ set. Elevation was measured as the mean elevation within a grid cell from a digital elevation model of approximately 1 km × 1 km resolution (the Global 30 Arc Second Elevation Data Set, http://www1.gsi.go.jp/geowww/globalmap-gsi/gtopo30/gtopo30.html). Following van Rensburg et al. [49], we considered a grid cell to be on a biome edge if a biome (as delineated by Olson et al. [61]) covering ≥5% of that grid cell also covered <5% of any of the neighboring grid cells. The number of biome edges was then calculated as the number of biomes in that grid cell meeting this definition.

To evaluate the overall relationships between β_sim-d_ and elevation and between β_sim-d_ and number of biome boundaries within a grid cell, we calculated the correlation between β_sim-d_ for the three taxa and each environmental variable. Correlations were calculated with Spearman rank correlation coefficients to accommodate the non-normal distributions of β_sim-d_. Standard significance tests are not appropriate for autocorrelated data because the assumption of independence is violated; therefore, we tested for significance using a method developed by Clifford et al. [65] that corrects the sample size of two variables based on the level of the spatial dependency in and between them [18]. We calculated the ess for each pair of variables using the PASSAGE software package [66], and then used the corrected degrees of freedom to test the significance of each correlation.

Pair-wise congruence at the hemisphere and biogeographic realm extents was measured as the correlation in β_sim-d_ values for each pair of taxa, and significance was tested using the method described above. To examine congruence at extents smaller than a biogeographic realm, we calculated the correlation in β_sim-d_ values within a ≤350-km-radius window (centroid to centroid distance) around each grid cell. We used this window size because it provided a better representation of the geographic variation in β_sim-d_ at small extents than the other window sizes we experimented with (radii of 150, 250, and 450 km). The same overall pattern was also apparent using larger windows but became increasingly muted as the extent widened. Moreover, larger windows had a greater discrepancy in the number of grid cells occurring within windows around coastal versus inland grid cells, while smaller windows considerably decreased the number of grid cells across which congruence was measured. The ≤350-km window was not substantially affected by either of these issues, and differences that did exist in the number of grid cells within coastal and interior windows did not appear to influence the geographical variation in congruence.

Spatial overlap between matching percentage sets of the highest 2.5% and lowest 2.5% of β_sim-d_ grid cells for each pair of taxa and for all three groups was calculated as the maximum overlap possible [34]: *N*_c_/*N*_t_, where *N*_c_ is the number of grid cells common to the sets and *N*_t_ is the total number of grid cells in the smallest set (amphibians have slightly fewer grid cells than birds or mammals).

## Supporting Information

Figure S1 Scatter Plots Showing Relationships between Beta-Diversity and Two Environmental Variables (Elevation and Number of Biome Edges within Grid Cells) For each panel, untransformed (left plots) and transformed (right plots) values of β_sim-d_ (*y*-axis) against either grid cell elevation (*x*-axis, upper plots) or number of biome edges within grid cell (*x*-axis, lower plots). In each plot, the red dots represent the highest 2.5% of β_sim-d_ grid cells, and the purple dots show the lowest 2.5% of β_sim-d_ grid cells. (A) Amphibians. (B) Birds. (C) Mammals.
(472 KB PDF)

Figure S2 Bird Beta-Diversity Based on Both Breeding and Non-Breeding Ranges Beta-diversity (β_sim-d_) values are divided into 20 quantiles, represented by warm (higher β_sim-d_) to cool (lower β_sim-d_) colors. The scale accompanying the color ramp shows minimum, first quartile, median, third quartile, and maximum values of β_sim-d_.
(210 KB PDF)

Figure S3 Average Nearest-Neighbor Beta-Diversity of Amphibians, Birds, and Mammals Mapped Continuously across the Continental Western Hemisphere Average nearest-neighbor beta-diversity (β_sim_) values are divided into 20 quantiles, represented by warm (higher β_sim_) to cool (lower β_sim_) colors. The scale accompanying the color ramp shows minimum, first quartile, median, third quartile, and maximum values of β_sim_. Gray grid cells do not contain amphibian species. (A) Amphibians. (B) Birds. (C) Mammals.
(503 KB PDF)

Table S1 Correlations between Beta-Diversity (β_sim-d_) and Two Environmental Variables (Elevation and Number of Biome Edges of Grid Cells) (81 KB PDF)

Table S2 Mean Elevation and Mean Number of Biome Edges for Sets of the Highest 2.5% and Lowest 2.5% of Beta-Diversity Grid Cells (56 KB PDF)

Table S3 Correlations in Average Nearest-Neighbor Beta-Diversity (β_sim_) within the Western Hemisphere, Nearctic Realm, and Neotropical Realm (81 KB PDF)

## Abbreviations

ess: estimated sample size
