# Supplementary material for: Putting Beta-Diversity on the Map: Broad-Scale Congruence and Coincidence in the Extremes
Source: PLoS Biol. 2007 Oct 9;5(10):e272. doi: 10.1371/journal.pbio.0050272 (PMC2001212; doi:10.1371/journal.pbio.0050272)

McKnight et al. Figure S3

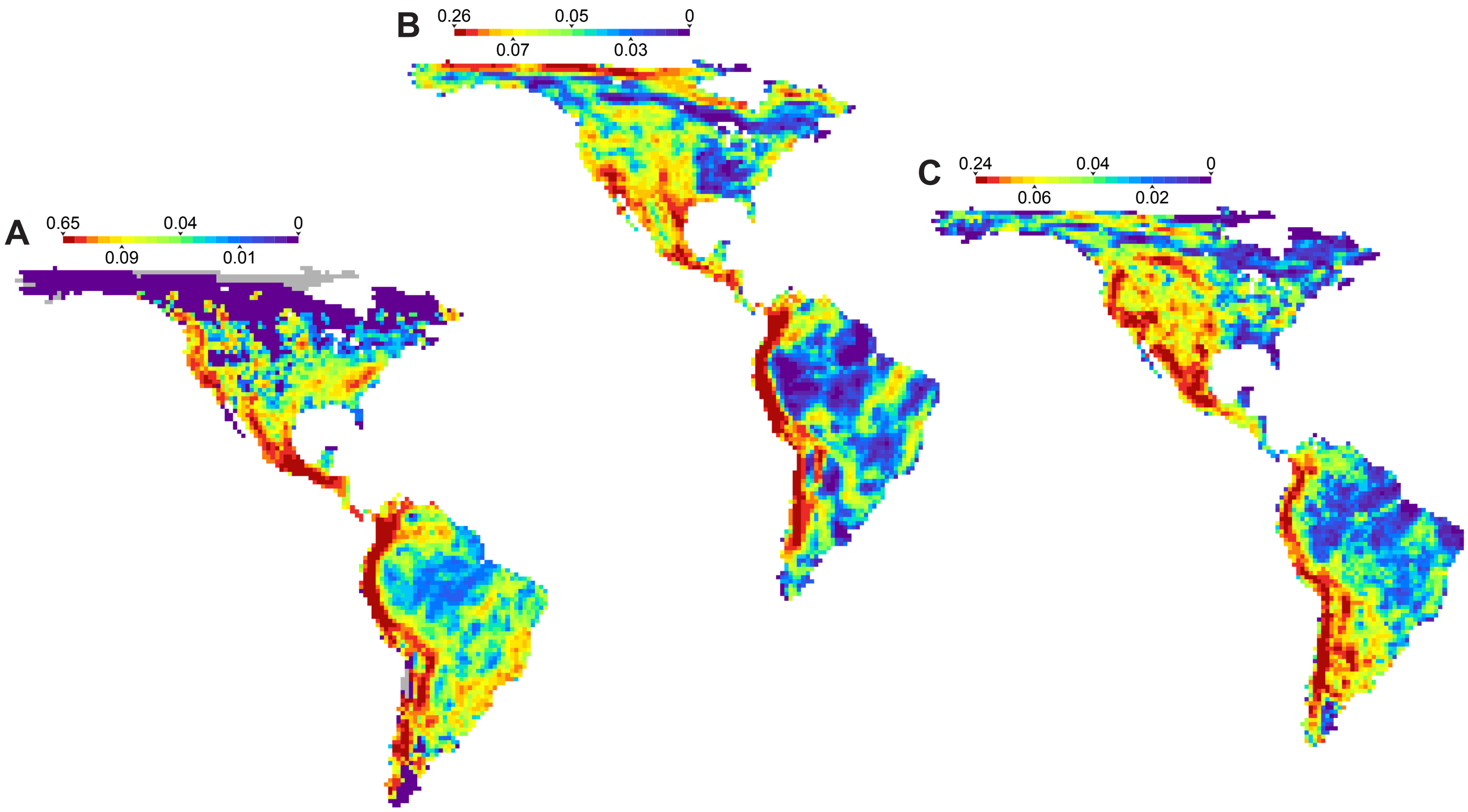

Supplement: Supplementary file 3 [file pbio.0050272.sg003.pdf]
