# Supplementary material for: Putting Beta-Diversity on the Map: Broad-Scale Congruence and Coincidence in the Extremes
Source: PLoS Biol. 2007 Oct 9;5(10):e272. doi: 10.1371/journal.pbio.0050272 (PMC2001212; doi:10.1371/journal.pbio.0050272)
Supplement: Supplementary file 4 [file pbio.0050272.st001.pdf]

Table S1. Correlations Between Beta-Diversity (β_{sim-d}) and Two Environmental Variables (Elevation and Number of Biome Edges within Grid Cells)

	Amphibian β_{sim-d}			Bird β_{sim-d}			Mammal β_{sim-d}		
	ρ	*n*	*ess*	*ρ*	*n*	*ess*	*ρ*	*n*	*ess*
Mean Elevation (m)	0.219*	3693	94.96	0.316**	3821	118.16	0.427**	3821	68.29
Number of Biome Edges	0.307**	3693	261.16	0.320**	3821	281.65	0.295**	3821	130.18

** $P < 0.001$, * $P < 0.05$

Spearman rank correlation coefficients (*ρ*), number of grid cells (*n*), and corrected sample size (*ess*) from each comparison are shown.
