# Supplementary material for: Putting Beta-Diversity on the Map: Broad-Scale Congruence and Coincidence in the Extremes
Source: PLoS Biol. 2007 Oct 9;5(10):e272. doi: 10.1371/journal.pbio.0050272 (PMC2001212; doi:10.1371/journal.pbio.0050272)
Supplement: Supplementary file 5 [file pbio.0050272.st002.pdf]

Table S2. Mean Elevation and Mean Number of Biome Edges for Sets of the Highest 2.5% and Lowest 2.5% of Beta-Diversity Grid Cells

	Low β_{sim-d} Sets	High β_{sim-d} Sets
Mean Elevation (m)		
Amphibians	480.49*	1681.36**
Birds	291.61**	1642.31**
Mammals	198.29**	1543.51**
Number of Biome Edges		
Amphibians	0.51**	1.72**
Birds	0.23**	1.44**
Mammals	0.32**	1.34**

** *P* <0.001, * *P* <0.05 (10,000 random sets of an equal number of grid cells)
