# Supplementary material for: Putting Beta-Diversity on the Map: Broad-Scale Congruence and Coincidence in the Extremes
Source: PLoS Biol. 2007 Oct 9;5(10):e272. doi: 10.1371/journal.pbio.0050272 (PMC2001212; doi:10.1371/journal.pbio.0050272)
Supplement: Supplementary file 6 [file pbio.0050272.st003.pdf]

Table S3. Correlations in Average Nearest-Neighbor Beta-Diversity (β_{sim}) within the Western Hemisphere, Nearctic Realm, and Neotropical Realm

	Amphibians and birds			Amphibians and mammals			Birds and mammals		
	ρ	*n*	*ess*	*ρ*	*n*	*ess*	*ρ*	*n*	*ess*
Western Hemisphere	0.325**	3,693	218.88	0.484**	3,693	99.49	0.512**	3,821	106.71
Nearctic Realm	0.103	1,744	90.33	0.417*	1,744	32.98	0.370*	1,862	59.01
Neotropical Realm	0.645**	1,878	87.28	0.596**	1,878	48.08	0.627**	1,888	46.19

** $P < 0.001$, * $P < 0.05$

Spearman rank correlation coefficients (*ρ*), number of grid cells (*n*), and corrected sample size (*ess*) from each pair-wise comparison are shown.
